# The Gut Microbiota Metabolite Urolithin B Mitigates Cholestatic Liver Injury in Mice via Modulating the Crosstalk Between PPARα, Nrf2, and NF-κB Signaling Pathways

**DOI:** 10.3390/jox15040128

**Published:** 2025-08-08

**Authors:** Hani M. Alrawili, Mahmoud Elshal, Marwa S. Serrya, Dina S. El-Agamy

**Affiliations:** Department of Pharmacology and Toxicology, Faculty of Pharmacy, Mansoura University, Mansoura 35516, Egypt

**Keywords:** cholestatic liver injury, urolithin B, oxidative stress and inflammation, Nrf2/NF-κB, PPARα, drug discovery

## Abstract

Urolithin (Uro)-B, a gut microbiota metabolite of ellagic acid, has recently gained considerable attention due to its beneficial bioactivities. This study investigated the potential hepatoprotective effect of Uro-B against alpha-naphthyl isothiocyanate (ANIT)-induced cholestatic liver injury (CLI) in mice and explored the possible involved mechanisms. Mice were treated with Uro-B (50 and 100 mg/kg) for four days and received ANIT (75 mg/kg) once on the second day. Our data revealed that Uro-B reduced elevated serum transaminases, alkaline phosphatase, lactate dehydrogenase, and total bilirubin levels associated with ANIT injection. Histopathologically, Uro-B effectively ameliorated ANIT-induced disruption of the hepatic architecture as represented by repressed necro-inflammation and bile duct proliferation. Uro-B also maintained oxidant/antioxidant status that was dysregulated by ANIT. Mechanistically, Uro-B markedly activated Kelch-like ECH-associated protein 1 (Keap-1)/nuclear factor erythroid 2-related factor 2 (Nrf2) signaling with subsequent upregulation of hepatic heme oxygenase-1 expression. On the other hand, Uro-B suppressed the ANIT-induced expression of nuclear factor kappa-B (NF-κB), tumor necrosis factor-alpha (TNF-α), and interleukin-6 (IL-6). Interestingly, Uro-B repressed peroxisome proliferator-activated receptor alpha (PPARα) expression in the liver. These findings indicate a promising hepatoprotective effect of Uro-B against ANIT-induced CLI in mice. Uro-B modulated the interplay between Keap1/Nrf2, NF-κB/TNF-α, and PPARα signaling pathways, resulting in powerful antioxidant and anti-inflammatory effects.

## 1. Introduction

Cholestasis is defined as decreased bile flow, which causes bile buildup in the liver. If this condition is not effectively controlled, it can lead to cholestatic liver injury (CLI) [1]. CLI has a complicated pathogenesis that involves oxidative stress and inflammatory responses. Reactive oxygen species (ROS) and other inflammatory mediators cause hepatocyte damage that impairs liver function and eventually hastens the progression of liver injury to liver fibrosis and cirrhosis [2].

The treatment approaches of cholestasis are still focused on the non-specific attenuation of cholestasis. Until now, ursodeoxycholic acid has been the standard drug for cholestasis treatment; however, its clinical effects are controversial [3]. Therefore, there is an urgent need to look for new candidate drugs that can mitigate the progression of cholestasis via the modulation of specific targets that control the pathogenesis of such liver injury.

The gut microbiota refers to the diverse microorganisms that inhabit the human gut. The gut microbiota coexists normally with humans in a healthy condition, allowing the host to keep normal biological function by releasing essential nutrients from food and controlling immunological responses. However, the gut microbiota composition and abundance are dynamic and influenced by different factors, including age, nutrition, and medications. There is accumulating evidence that dysregulated gut microbiota is linked to the initiation and progression of various liver diseases, such as CLI [4,5]. Urolithin (Uro)-B, a metabolite generated by the gut microbiota metabolism of ellagic acid, has recently gained considerable attention due to its possible therapeutic advantages. Many fruits and vegetables contain the polyphenol ellagic acid, which is bio-transformed by gut bacteria into Uro-B [6]. Uro-B has been associated with different biological activities, including antioxidants and anti-inflammatory ones [7,8].

Recent studies have demonstrated that Uro-B exerts antioxidant and anti-inflammatory effects in unilateral ureteral obstruction-induced kidney damage [9], Helicobacter pylori-induced inflammation and oxidative stress [10], and osteoarthritis [11]. These characteristics imply that Uro-B could offer a novel, safe, and effective treatment option in CLI, where oxidative stress and inflammation significantly underlie its development [12].

Alpha-naphthyl isothiocyanate (ANIT) is a strong toxin that has been widely used to establish a mouse model of CLI that is linked to oxidative stress and inflammation, simulating human CLI [13,14]. Accordingly, this study aimed to explore the ability of Uro-B to counteract ANIT-induced CLI in mice and explore its potential underlying mechanisms.

## 2. Materials and Methods

### 2.1. Drugs and Chemicals

Uro-B and ANIT were obtained from Sigma-Aldrich (St. Louis, MO, USA) and prepared in corn oil and 0.5% carboxymethyl cellulose (CMC), respectively. All other chemicals and supplementary reagents used in this study were purchased from reliable vendors and applied in compliance with the instructions provided by the manufacturer.

### 2.2. Animals

Male BALB/c albino mice weighing 25–35 g were supplied by VACSERA (Giza, Egypt). Mice were kept in a controlled setting with a 12 h light/dark cycle and a constant temperature of 25 °C. They freely have access to ordinary rodent food and water. The experimental protocol has been approved by the Mansoura University Animal Care and Use Committee (MU-ACUC, PHARM.PhD.23.05.23) and performed according to the U.K. Animals (Scientific Procedures) Act, 1986, and related guidelines. Reporting the animal experiment follows the ARRIVE guidelines.

### 2.3. Experimental Design

The mice were randomly assigned into five groups, each of five animals: (1) Control group: the mice were only given the vehicles; (2) Uro-B group: the mice received a dose of 100 mg/kg Uro-B for four successive days; (3) ANIT group: the mice were given a single intragastric injection of ANIT at a dose of 75 mg/kg to induce cholestatic liver damage [15]; (4) Uro-B50 + ANIT group: the mice were orally administered Uro-B (50 mg/kg) for four successive days and a single ANIT injection two hours following the second dose of Uro-B; (5) Uro-B100 + ANIT group: the mice were orally given Uro-B (100 mg/kg) for four successive days and a single ANIT injection two hours following the second Uro-B dose. The doses of Uro-B were selected based on our pilot study and a previous study by Chen et al. [16]. The administration of Uro-B prior to ANIT was designed to evaluate its potential hepatoprotective effect by allowing sufficient time for the compound to modulate hepatic antioxidant and anti-inflammatory pathways before exposure to the hepatotoxic insult.

Two days after receiving the ANIT, the mice were anesthetized using thiopental (70 mg/kg, i.p.) [17]. Blood samples were taken by heart puncture, and sera were isolated for subsequent biochemical analysis. Additionally, livers were removed and cut into four parts: The first part was kept in 10% neutral buffered formalin for further preparation of paraffin blocks and histological and immunohistochemical analyses. The second part was used for preparing liver homogenates. Otherwise, the third and fourth parts were kept for further RT-PCR and Western blot analysis.

### 2.4. Assessment of Liver Function

Serum levels of liver injury indicators, including alanine aminotransferase (ALT), aspartate aminotransferase (AST), lactate dehydrogenase (LDH), alkaline phosphatase (ALP), and total bilirubin (TBIL), were measured to evaluate liver function using commercially available kits obtained from HUMAN Diagnostics (Wiesbaden, Germany) following the manufacturer’s instructions.

### 2.5. Histopathological Evaluation of Hepatic Necroinflammation and Bile Duct Injury

The paraffin-embedded liver tissues were sectioned into 4–5 µm thick pieces, stained with hematoxylin-eosin (H&E), and investigated under a light microscope by a pathologist in a blind manner. Neuroinflammatory changes were assessed and graded as previously described by Ishak et al. [18]. In addition, a bile duct injury severity score (BDISS) was used to semi-quantify bile duct injury in liver biopsies. This score was based on three components: bile duct damage, ductular proliferation, and cholestasis, where each was graded as 0, absent; 1, mild; 2, moderate; 3, severe, using a modified version of the Banff criteria [19].

### 2.6. Assessment of Hepatic Oxidative Stress Biomarkers

Malondialdehyde (MDA), reduced glutathione (GSH), superoxide dismutase (SOD), and total antioxidant capacity (TAC) levels in the liver were measured to assess hepatic redox status. These biomarkers are precisely quantified by specific kits from Bio-Diagnostic (Giza, Egypt).

### 2.7. Western Blot Analysis

In brief, total protein was extracted from each sample using the ReadyPrep^TM^ protein extraction kit (Bio-Rad Inc., Hercules, CA, USA) and then determined using the Bradford protein assay kit (Bio Basic Inc., Markham, ON, Canada). Equivalent samples (20 μg proteins) using 2× Laemmli sample buffer were heated to 95 °C for five minutes to ensure protein denaturation before loading into polyacrylamide gel electrophoresis. The TGX Stain-Free^TM^ FastCast^TM^ Acrylamide Kit (SDS-PAGE), supplied by Bio-Rad Inc., was used. The gel was put in a transfer sandwich as follows: filter paper, PVDF membrane, gel, and filter paper. The sandwich was added to 1× transfer buffer (190 mM glycine, 20% methanol, and 25 mM Tris) in the transfer tank. The Bio-Rad Trans-Blot Turbo was then used to run the blot for seven minutes at 25 V for one hour. After that, the membrane was blocked in tris-buffered saline tween 20 (TBST) and 3% bovine serum albumin at room temperature. The membrane was then incubated with nuclear factor erythroid 2-related factor 2 (Nrf2) and heme oxygenase-1 (HO-1), Kelch-like ECH-associated protein 1 (Keap-1), and NAD(P)H quinone dehydrogenase 1 (NQO1) primary antibodies (Cell Signaling, Danvers, MA, USA) at 4 °C overnight on a shaker, followed by the HRP-conjugated secondary anti-rabbit IgG (Novus Biologicals, LLC) at room temperature for one hour. A chemiluminescent substrate (Clarity^TM^ Western ECL substrate, Bio-Rad) was utilized for visualization. A CCD camera-based imager and image analysis software were used to read the band intensity of the proteins of interest against β-actin as a reference protein.

### 2.8. Enzyme-Linked Immunosorbent Assay (ELISA) Measurements

Commercially available ELISA kits were used to determine the hepatic levels of Nrf2 and HO-1 (Cloud-Clone Corp., Houston, TX, USA), tumor necrosis factor-alpha (TNF-α) and interleukin-6 (IL-6) (Fine Test, Wuhan, China), and peroxisome proliferator-activated receptor alpha (PPARα) (Sino Biological, Houston, TX, USA) according to the manufacturer’s instructions.

### 2.9. Immunohistochemical (IHC) Analysis

IHC staining was performed using the Ventana-Bench-Mark-XT system (Ventana Medical Systems, Tucson, AZ, USA) as previously described [20]. Briefly, hepatic sections were immuno-stained through incubation with specific primary antibodies against NF-κB p65 (ABclonal, Wobum, MA, USA), Nrf2 (Diagnocine, Hackensack, NJ, USA), and proliferating cell nuclear antigen, PCNA (BioLegend, London, UK), overnight at 4 °C. Sections were incubated with HRP-conjugated anti-rabbit secondary antibody (Novus Biologicals, LLC, Centennial, CO, USA) at room temperature. Immuno-stained specimens were visualized through incubation at room temperature with diaminobenzidine for 5 min and hematoxylin for 1 min as a counterstain. The area percent of the positive immunoreactivity was estimated by image analysis software (ImageJ, version 1.53t, NIH) after color deconvolution.

### 2.10. Real-Time Reverse Transcription-Polymerase Chain Reaction (RT-PCR)

Following the manufacturer’s instructions, RNA was extracted from liver samples using Direct-zol RNA Miniprep Plus (Zymo Research Corp., Irvine, CA, USA). Superscript IV One-Step RT-PCR kit (Thermo Fisher Scientific, Waltham, MA, USA) was used to reverse transcribe the extracted RNA and then perform PCR in a single step. Step One RT-PCR (Applied BioSystems, Waltham, CA, USA) was used to apply the prepared reaction mixture samples in real time. Within the same sample and using the corresponding primers, the mRNA levels of Nrf2 (forward: TGTGGAGAGTGGGAGCCTCT and reverse: CCTGGCTGGTGATGAGGATT), HO-1 (forward: AGCAGCTGGGATGATGGGAC and reverse: TGGCTGCTGGGATTGGAATG), NQO1 (forward: AGCCACAGGTCGAGAGCTGA, and reverse: TGGTGGAAGCAGTAGCGGAC), and PPARα (forward: TCTGCAGCAGGCTCTGTTGC and reverse: GAGGGGTTGTCCAGGAAGAG) were standardized in relation to glyceraldehyde-3-phosphate dehydrogenase (Gapdh, forward: TTGTGCAGTGCCAGCCTCGT and reverse: TGCCGTTGAACTTGCCGTGG). Relative mRNA expression of genes of interest was determined through the ΔΔCt method.

### 2.11. Statistical Analysis

Statistical analysis was performed using GraphPad Prism V8.01 (GraphPad Software Inc., San Diego, CA, USA). Data are expressed as mean ± standard deviation (SD). For parametric data, one-way ANOVA and the subsequent Tukey–Kramer multiple comparisons test were applied. For non-parametric data, we used the Kruskal–Wallis test followed by the Dunn post hoc test. A *p* < 0.05 was set as statistical significance.

## 3. Results

### 3.1. Impact of Uro-B on Liver Function Biomarkers in ANIT-Challenged Mice

**Figure 1:**
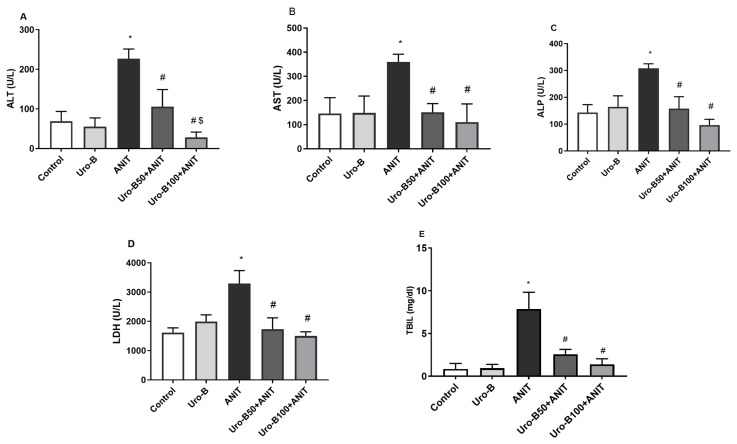
Effect of urolithin B (Uro-B, 50 and 100 mg/kg) on liver function parameters. (**A**) Alanine aminotransferase (ALT), (**B**) Aspartate aminotransferase (AST), (**C**) Alkaline phosphatase (ALP), (**D**) Lactate dehydrogenase (LDH), and (**E**) total bilirubin (TBIL) serum levels in α-naphthyl isothiocyanate (ANIT)-injected mice. Data are expressed as mean ± SD (n = 5). * *p* < 0.05 compared to the control group; ^#^ *p* < 0.05 compared to the ANIT group; ^$^ *p* < 0.05 compared to the Uro-B50 + ANIT group using one-way ANOVA followed by the Tukey–Kramer multiple comparisons post hoc test.

Following the ANIT challenge, the levels of serum ALT, AST, ALP, LDH, and TBIL in the ANIT group significantly increased by about 3.27-, 2.46-, 2.14-, 2-, and 9.2-fold, respectively, in comparison to the control group (Figure 1A–E, respectively). Meanwhile, oral administration of Uro-B (50 and 100 mg/kg) significantly reduced serum levels of ALT (by about 53% and 88%, respectively), AST (by about 58% and 69%, respectively), ALP (by about 49% and 69%, respectively), LDH (by about 47% and 54%, respectively), and TBIL (by about 67% and 82%, respectively) in comparison to the ANIT group.

### 3.2. Impact of Uro-B on Hepatic Histopathological Changes in ANIT-Challenged Mice

**Figure 2:**
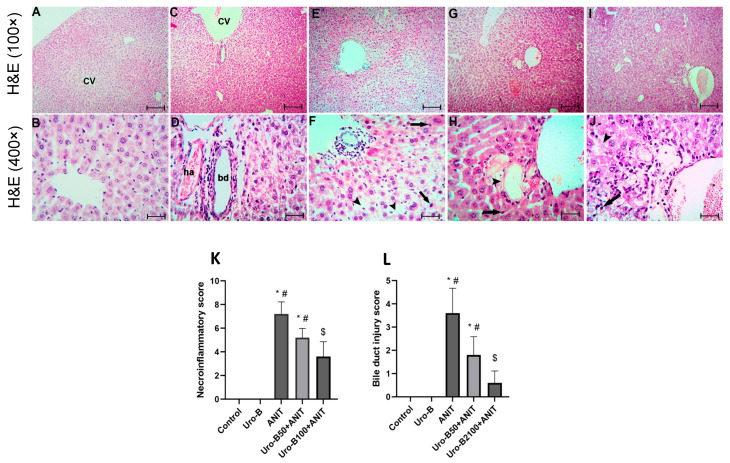
Effect of urolithin B (Uro-B, 50 and 100 mg/kg) on hepatic histopathological changes illustrated using hematoxylin-eosin (H&E) stain. (**A**,**B**) Hepatic sections of the control group show a normal liver triad [formed of the central vein (CV), hepatic arteriole (ha), and bile ductule (bd)] and separated by sinusoidal space with apparently healthy hepatocytes arranged in plates radiating from the triad and separated by sinusoidal space. (**C**,**D**) Hepatic sections of the Uro-B group show more or less as the control group. (**E**,**F**) Hepatic sections of the ANIT group show congestion of the CVs, inflammatory cell infiltration (arrow) and vacuolation of the cytoplasm of the hepatocytes (arrow heads). (**G**,**H**) Hepatic sections of the Uro-B 50 + ANIT group show non-congested CVs, less marked inflammatory cell infiltration (arrow) and vacuolation of the cytoplasm of the hepatocytes. (**I**,**J**) Hepatic sections of the Uro-B100 + ANIT group show absence of inflammatory cell infiltration and minimal vacuolation of the cytoplasm of the hepatocytes (**A**,**C**,**E**,**G**,**I**) × 100, bar = 400 µm; (**B**,**D**,**F**,**H**,**J**) × 400, scale bar = 25 µm., (**K**) Corresponding necroinflammatory scoring, and (**L**) Corresponding bile duct injury score. Data are expressed as mean ± SD (n = 5). * *p* < 0.05 compared to the control group; ^#^ *p* < 0.05 compared to the ANIT group; ^$^ *p* < 0.05 compared to the Uro-B50 + ANIT group using Kruskal–Wallis followed by Dunn’s multiple comparison test.

Histopathological examination of the liver tissues from the ANIT group (Figure 2E,F) revealed widespread hepatic architectural disruption, bile duct destruction, and marked necroinflammation, while normal hepatic architecture was present in the control (Figure 2A,B) and Uro-B (Figure 2C,D) groups. Treatment with Uro-B markedly mitigated these hepatic histological lesions. Necroinflammation and bile duct injury were greatly suppressed in the lower and higher Uro-B-treated groups, Uro-B50 + ANIT (Figure 2G,H) and Uro-B100 + ANIT (Figure 2I,J); however, the higher dose provided more protection. In addition, semi-quantitative analysis was performed and revealed that the ANIT challenge significantly increased the scores of necroinflammation (Figure 2K) and bile duct injury (Figure 2L) compared to the control group, whereas the higher dose of Uro-B significantly reduced these scores.

### 3.3. Impact of Uro-B on Hepatic Redox Status in ANIT-Challenged Mice

**Figure 3:**
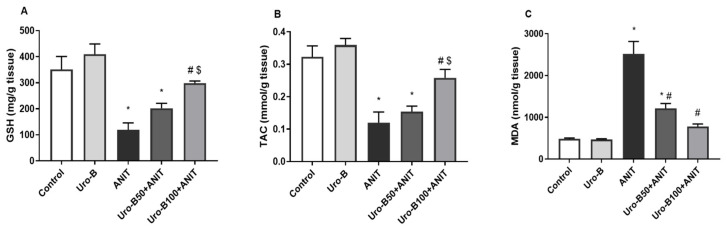
Effect of urolithin B (Uro-B, 50 and 100 mg/kg) on oxidative stress parameters. (**A**) Malondialdehyde (MDA), (**B**) reduced glutathione (GSH), and (**C**) total antioxidant capacity (TAC) in hepatic tissue of α-naphthyl isothiocyanate (ANIT)-injected mice. Data are expressed as mean ± SD (n = 5). * *p* < 0.05 compared to the control group; ^#^ *p* < 0.05 compared to the ANIT group; ^$^ *p* < 0.05 compared to the Uro-B50 + ANIT group using one-way ANOVA followed by the Tukey–Kramer multiple comparisons post hoc test.

Following the ANIT challenge, the levels of MDA, the lipid peroxidation biomarker, in the liver significantly increased by about 5-fold (Figure 3A). However, the hepatic levels of the antioxidants GSH and TAC were significantly reduced by about 66% and 63%, respectively, in comparison to the control group (Figure 3B and Figure 3C, respectively). Meanwhile, oral administration of Uro-B (50 and 100 mg/kg) significantly decreased the MDA levels by about 52% and 69%, respectively, compared to the ANIT group, showing a dose-related effect. Only the higher Uro-B dose (100 mg/kg) was able to significantly increase the hepatic GSH and TAC levels by about 2.5- and 2.15-fold, respectively. Notably, there was no significant difference between the control and Uro-B groups regarding either liver function biomarkers, histopathological scores, or oxidative stress biomarkers; hence, we completed our study on the control, ANIT, Uro-B50 + ANIT, and Uro-B100 + ANIT groups.

### 3.4. Impact of Uro-B on Hepatic Keap-1, Nrf2, HO-1, and NQO1 Expression in ANIT-Challenged Mice

**Figure 4:**
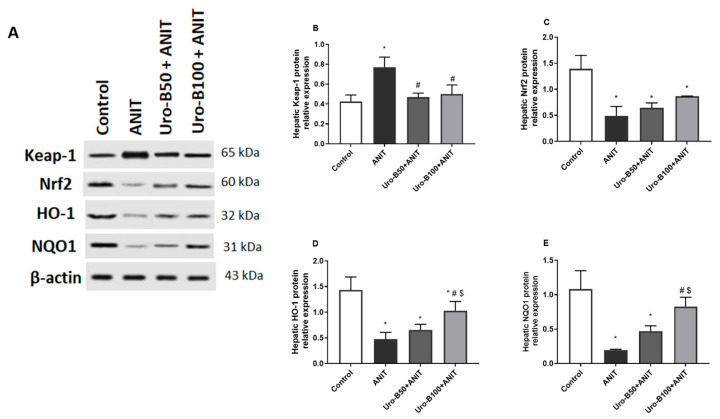
(**A**) Representative Western blot images demonstrating the effect of urolithin B (Uro-B, 50 and 100 mg/kg) on the protein expression of Kelch-like ECH-associated protein 1 (Keap-1), Nuclear factor erythroid 2-related factor 2 (Nrf2), Heme oxygenase 1 (HO-1), NAD(P)H dehydrogenase (quinone) 1 (NQO1), and the house-keeping protein β-actin in α-naphthyl isothiocyanate (ANIT)-injected mice. (**B**–**E**) Corresponding relative protein expression levels of Keap1, Nrf2, HO-1, and NQO1 in the liver (n = 3). Data are expressed as mean ± SD. * *p* < 0.05 compared to the control group; ^#^ *p* < 0.05 compared to the ANIT group; ^$^ p < 0.05 compared to the Uro-B50 + ANIT group using one-way ANOVA followed by Tukey–Kramer multiple comparisons post hoc test.

When cholestasis occurs, the liver is particularly vulnerable to oxidative burden due to the imbalance between ROS production and antioxidant defense [21]. Nrf2 is an important nuclear transcription factor that controls the expression of important antioxidants. Our Western blotting results (Figure 4A) revealed that the ANIT injection significantly increased hepatic Keap1 protein expression by about 1.8-fold, whereas it significantly decreased Nrf2, HO-1, and NQO1 protein expression levels in the liver (by about 64%, 67%, and 82%, respectively) in comparison to the control group (Figure 4B–E, respectively). Meanwhile, hepatic Keap1 protein expression levels were significantly suppressed upon oral administration of Uro-B (50 and 100 mg/kg) by about 39% and 35%, respectively. Alternatively, the lower and higher doses of Uro-B elevated the hepatic protein levels of Nrf2, HO-1, and NQO1, but the effect was only significant with the higher dose of Uro-B on NQO1 levels that were elevated by about 4.2-fold, compared to the ANIT group.

**Figure 5:**
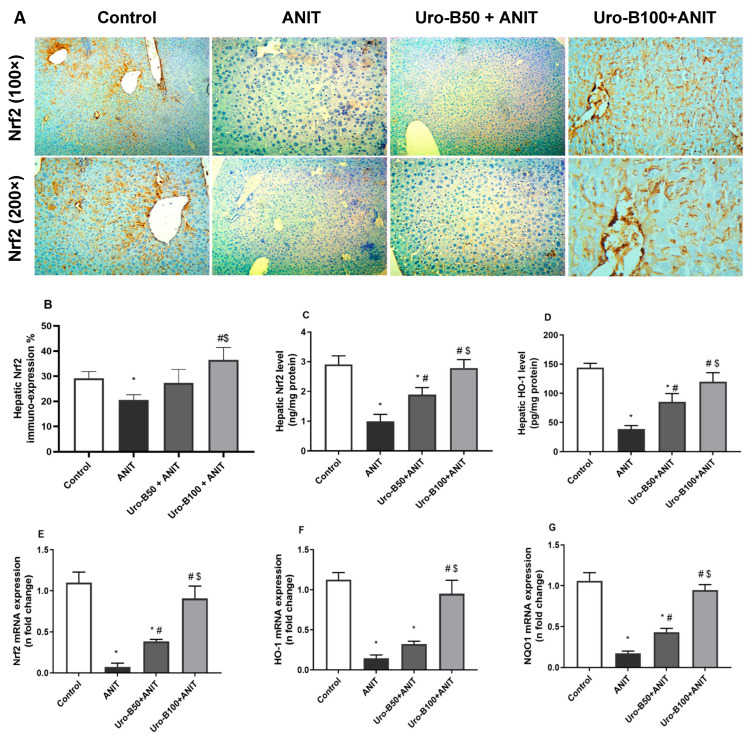
(**A**) Representative immuno-stained hepatic sections showing the effect of urolithin B (Uro-B, 50 and 100 mg/kg) on the nuclear factor erythroid 2-related factor 2 (Nrf2) expression in α-naphthyl isothiocyanate (ANIT)-injected mice, (**B**) corresponding area percent of the positive Nrf2 immunoreactivity, (**C**,**D**) effect of Uro-B on Nrf2 and heme oxygenase (HO-1) protein levels, respectively, measured by ELISA, (**E**–**G**) effect of Uro-B on Nrf2, HO-1, and NAD(P)H dehydrogenase quinone 1 (NQO1) relative gene expression levels in the liver. Data are expressed as mean ± SD (n = 5). * *p* < 0.05 compared to the control group; ^#^ *p* < 0.05 compared to the ANIT group; ^$^ *p* < 0.05 compared to the Uro-B50 + ANIT group, using one-way ANOVA followed by the Tukey–Kramer multiple comparisons post hoc test.

The immuno-expression of Nrf2 in the liver was further investigated and represented in Figure 5A. The ANIT challenge resulted in a significant decline in Nrf2 expression in the liver (Figure 5B), indicated by brown cytoplasmic coloration only in a few hepatocytes. Noticeably, the higher dose of Uro-B (100 mg/kg) significantly increased Nrf2 hepatic expression, as indicated by dense brown staining of hepatocytes.

In addition, the changes in Nrf2 and HO-1 protein expression levels in the liver were further addressed by ELISA. Our results revealed that the ANIT challenge significantly decreased serum levels of Nrf2 and HO-1 by about 65% and 73%, respectively, compared to the control group (Figure 5C and Figure 5D, respectively). In comparison with the ANIT group, oral administration of Uro-B (50 and 100 mg/kg) significantly increased the hepatic levels of Nrf2 (by about 1.89- and 2.79-fold) and HO-1 (by about 2.2- and 3.1-fold, respectively).

Moreover, the impact of Uro-B on the hepatic Nrf2, HO-1, and NQO1 expression at the gene level was addressed. Our data showed that the ANIT challenge was associated with a significant decrease in the hepatic relative gene expression of Nrf2, HO-1, and NQO by about 93%, 87%, and 83%, respectively, compared to the control group (Figure 5E–G). Meanwhile, oral administration of Uro-B (50 mg/kg) significantly increased hepatic relative gene expression of Nrf2 and NQO1 by about 5.2- and 2.5-fold, respectively, compared to the ANIT group. Otherwise, the higher dose of Uro-B (100 mg/kg) caused a significant increase in Nrf2, HO-1, and NQO1 by about 12.3-, 6.5-, and 5.5-fold, respectively, compared to the ANIT group.

### 3.5. Impact of Uro-B on Hepatic NF-κB, TNF-α, and IL-6 Expression in ANIT-Challenged Mice

**Figure 6:**
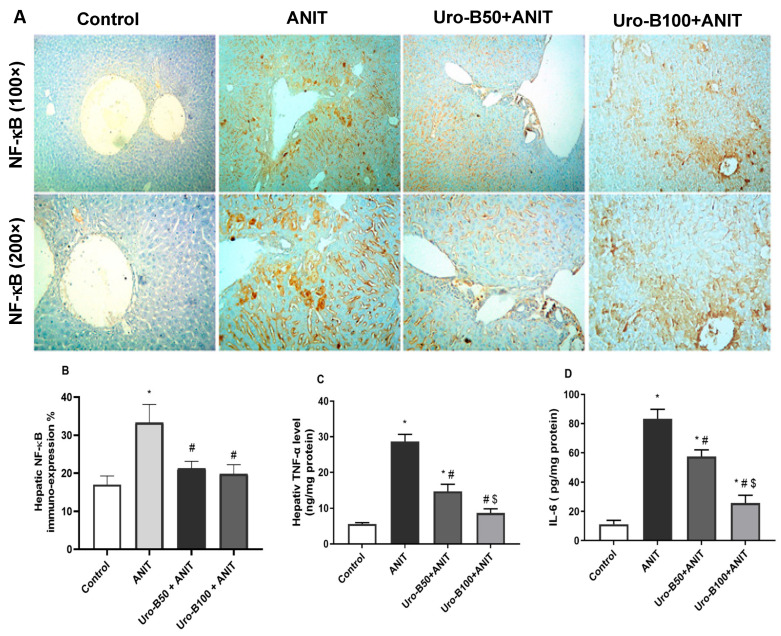
(**A**) Representative immuno-stained hepatic sections showing the effect of urolithin B (Uro-B, 50 and 100 mg/kg) on the nuclear factor kappa-B (NF-κB) p65 expression in α-naphthyl isothiocyanate (ANIT)-injected mice. (**B**) Corresponding area percent of the positive NF-κB p65 immunoreactivity. (**C**,**D**) Effect of Uro-B on tumor necrosis factor alpha (TNF-α) and interleukin (IL)-6 protein levels, respectively, measured by ELISA. Data are expressed as mean ± SD (n = 5). * *p* < 0.05 compared to the control group; ^#^ *p* < 0.05 compared to the ANIT group; ^$^ *p* < 0.05 compared to the Uro-B50 + ANIT group, using one-way ANOVA followed by Tukey–Kramer multiple comparisons post hoc test.

ANIT-induced hepatotoxicity was reported to cause neutrophil inflammation around the bile duct and portal tract, resulting in a considerable hepatic inflammatory reaction [22]. NF-κB is a primary mediator that transfers the inflammatory signals from the cytoplasm to the nucleus and further initiates the inflammatory signaling cascade in the cell, such as TNFα, IL-6, and IL-1β [23,24]. As demonstrated in Figure 6A, ANIT led to a considerable rise in NF-κB p65 expression, as indicated by intense immunopositive brown staining of hepatocytes that was markedly mitigated by Uro-B administration. This was reflected in the significant increase in NF-κB p65 expression percentage in the ANIT group compared to the control group (Figure 6B). Otherwise, this expression percentage was significantly decreased by Uro-B (50 and 100 mg/kg) administration in comparison with the ANIT group.

Regarding TNF-α and IL-6, our ELISA results revealed that ANIT significantly increased their hepatic levels by about 5- and 7.4-fold, respectively, compared to the ANIT group (Figure 6C and Figure 6D, respectively). Meanwhile, oral administration of Uro-B (50 and 100 mg/kg) significantly decreased these hepatic levels of TNF-α (by about 48.55% and 69.72%, respectively) and IL-6 (by about 30.94% and 69.17%, respectively) in comparison with the ANIT group.

### 3.6. Impact of Uro-B on Hepatic PPARα Expression in ANIT-Challenged Mice

**Figure 7:**
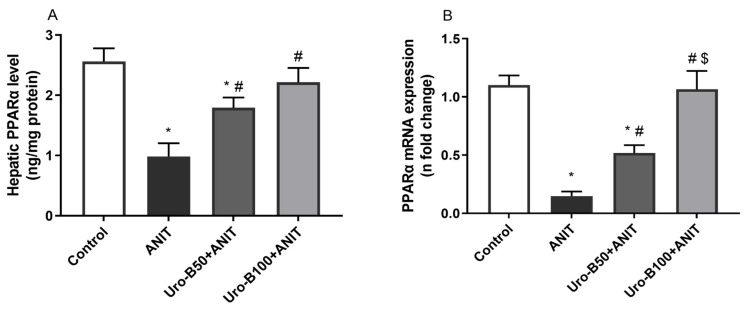
(**A**,**B**) Effect of urolithin B (Uro-B, 50 and 100 mg/kg) on peroxisome proliferator-activated receptor alpha (PPARα) protein and relative gene expression levels, respectively, in α-naphthyl isothiocyanate (ANIT)-injected mice. Data are expressed as mean ± SD (n = 5). * *p* < 0.05 compared to the control group; ^#^ *p* < 0.05 compared to the ANIT group; ^$^ *p* < 0.05 compared to the Uro-B50 + ANIT group using one-way ANOVA followed by the Tukey–Kramer multiple comparisons post hoc test.

PPARα activation protects against CLI by controlling bile acid homeostasis [25]. Compared to the control mice, ANIT-injected mice had significantly lower hepatic levels of PPARα by about 61% (Figure 7A). Interestingly, both Uro-B doses (50 and 100 mg/kg) significantly increased PPARα levels by about 1.8- and 2.25-fold, respectively, compared to the ANIT group.

Moreover, the changes in PPARα gene expression in the liver were addressed, and our data revealed that ANIT-induced CLI is associated with a significant decrease in the hepatic gene expression of PPARα by about 86.5% when compared to the control group (Figure 7B). Otherwise, oral administration of Uro-B (50 and 100 mg/kg) significantly increased its gene expression by about 3.5- and 7.2-fold, respectively, compared to the ANIT group.

### 3.7. Impact of Uro-B on Hepatic PCNA Expression in ANIT-Challenged Mice

**Figure 8:**
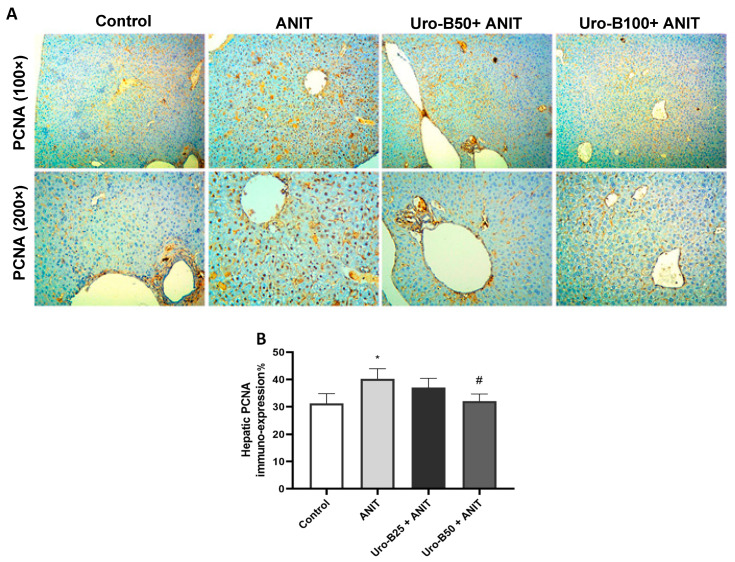
(**A**) Representative immuno-stained hepatic sections showing the effect of urolithin B (Uro-B, 50 and 100 mg/kg) on the proliferating cell nuclear antigen (PCNA) expression in α-naphthyl isothiocyanate (ANIT)-injected mice. (**B**) Corresponding area percent of the positive PCNA immunoreactivity. Data are expressed as mean ± SD (n = 5). * *p* < 0.05 compared to the control group; ^#^ *p* < 0.05 compared to the ANIT group, using one-way ANOVA followed by Tukey–Kramer multiple comparisons post hoc test.

ANIT administration leads to a considerable increase in the cholangiocyte proliferation marker, PCNA [26]. A considerable rise in PCNA immuno-expression was addressed in the ANIT group, as represented in Figure 8A, which was attenuated by Uro-B administration, particularly by the higher dose (50 mg/kg). As demonstrated in Figure 8B, the ANIT group had significantly higher levels of PCNA expression compared to the control group. Intriguingly, treatment with Uro-B (100 mg/kg) was able to significantly repress these levels.

## 4. Discussion

The findings of the current study show the hepatoprotective effect of Uro-B in a mouse model of cholestatic liver damage triggered by ANIT via halting oxidative stress and inflammation in the liver. Herein, the ANIT injection led to a significant elevation in ALT, AST, ALP, LDH, and TBIL serum levels, indicating hepatocyte damage and liver dysfunction. A major decrease in these levels was observed with the administration of Uro-B, implying a beneficial effect on liver function. The effectiveness of Uro-B in minimizing liver injury is additionally demonstrated by the ability to ameliorate the liver architecture revealed upon histopathological evaluation, where Uro-B decreased necroinflammation and bile duct injury.

Oxidative stress underlies the pathogenesis of liver damage of different etiologies, including cholestasis, where the buildup of toxic bile acids can cause lipid peroxidation and the release of ROS [13,27]. This study revealed that ANIT administration increased hepatic MDA levels, a lipid peroxidation indicator, while reducing GSH levels and TAC, crucial for hindering oxidative stress. Uro-B successfully decreased MDA levels and increased GSH and TAC levels, especially with the higher dose. These results imply that Uro-B has strong antioxidant activity. The ability of Uro-B to counteract oxidative damage was previously reported by Li et al. [28], who demonstrated that Uro-B attenuated oxidative stress in a rat model of cerebral ischemia–reperfusion injury.

The transcription factor Nrf2 is generally known to enhance the cellular antioxidant defense system and, hence, reduce oxidative injury. Under no oxidative stimuli, Nrf2 exists in the cytoplasm sequestered by the Keap1 complex that mediates Nrf2 ubiquitination and subsequent proteasomal degradation. Upon exposure to electrophilic/oxidative stress, the complex Keap1 is dissociated, leading to the accumulation of Nrf2, which translocates into the nucleus and binds to the antioxidant response element, promoting the transcription of antioxidant gene that encode redox balancing factors, detoxifying enzymes, and stress response proteins, such as GSH, GST, NQO1, HO-1, and glutathione cysteine ligase catalytic subunit [29,30,31]. HO-1 and NQO1 expression reduce oxidative stress by facilitating removal of ROS [32]. Recent data indicate that the Nrf2 antioxidant pathway is pivotal in ANIT-induced cholestatic liver damage, where activation of the Nrf2 signaling pathway is associated with antioxidant and anti-inflammatory responses, as well as regulation of bile acid metabolism [33,34]. Consequently, Nrf2 is a prospective target for the treatment of cholestatic liver damage.

On the other hand, the transcription factor NF-κB is a master regulator of inflammation that controls the production of different inflammatory cytokines, including TNF-α and IL-6 [35]. A crosstalk between NF-κB and Nrf2 exists, where both factors are redox-sensitive. NF-κB is activated under oxidative stress, and the absence of Nrf2 leads to heightened oxidative stress and exacerbated NF-κB activity, resulting in an increase in inflammatory cytokine production [36,37].

Previous studies revealed suppressed Nrf2 expression and upregulated Keap1, NF-κB, TNF-α, and IL-6 expression during ANIT-induced cholestatic liver damage in mice [38,39]. It is worth mentioning that the reduced expression of Nrf2 and increased expression of the repressor Keap1 under ANIT-induced hepatotoxicity may be due to the associated severe oxidative stress that can paradoxically suppress Nrf2 expression and activity, possibly due to excessive activation of Keap1-mediated degradation or impaired nuclear translocation of Nrf2 [21,33,40].

In line with these studies, the present study demonstrated a significant decrease in the hepatic gene and protein levels of Nrf2, HO-1, and NQO1 in addition to a significant rise in Keap1, NF-κB, TNF-α, and IL-6 levels after ANIT injection. Otherwise, Uro-B intervention led to a considerable increase in Nrf2, HO-1, and NQO1 gene and protein expression levels and caused a significant drop in Keap1, NF-κB, TNF-α, and IL-6 hepatic levels. These results are consistent with the previous one that indicated the antioxidant and anti-inflammatory effects of Uro-B in lipopolysaccharide-injected mice, mainly via boosting Nrf2 signaling while suppressing NF-κB signaling [41]. Recent studies pointed to the role of Uro-B in the activation of Nrf2. Uro-B maintained renal oxidant/antioxidant balance in 5-fluorouracil-induced nephrotoxicity through enhancing Nrf2/HO-1 antioxidant protective responses with subsequent suppression of NF-κB inflammatory signaling [28,42]. Furthermore, Uro-B effectively inhibited oxidative stress induced by cerebral ischemia–reperfusion injury through the activation of the Nrf2/HO-1 signaling pathway [43]. Collectively, these results confirmed the potential role of Uro-B in sequestering ROS through activation of the Nrf2 signaling pathway which may be responsible in part for its hepatoprotective activity.

PPARs are nuclear receptors that play a key role in regulating the expression of different antioxidants and pro-inflammatory genes, rendering them among the most promising targets in oxidative stress-mediated inflammatory conditions. All PPAR subtypes are well-known to have an anti-inflammatory effect that occurs via various mechanisms [44]. An important crosstalk between Nrf2 and PPARs exists. PPARα is directly involved in the upregulation of antioxidant and anti-inflammatory gene expression, including HO-1 [45]. Moreover, PPARs directly interact with the p65 component of NF-κB, inhibiting its transcriptional activity. PPARα activation has been reported to further halt the NF-κB pathway through the targeting of the inhibitor kappa B-alpha [46]. PPARα is involved in the regulation of bile acid metabolism, and the PPARα-null mice demonstrated more aggressive toxic effects of ANIT-induced cholestasis compared to wild-type mice, indicating a protective potential of basal PPARα [47,48,49]. Recently, PPARα has been reported as a new therapeutic target to control the synthesis and transport of bile acids and inflammatory response during cholestasis, reducing cholestatic liver damage [50].

Compatible with these previous reports, ANIT injection in this study led to a significant reduction in PPARα hepatic levels, an indication of bile acid accumulation. In contrast, administration of Uro-B significantly restored both protein and gene expression levels of hepatic PPARα, suppressing cholestasis-mediated liver injury. The positive effect of Uro-B on PPARα expression has been stated before in high-fat diet-induced obesity in rats [51].

In many cases, bile duct growth is a hallmark of cholestatic diseases. Cholangiocytes proliferate in response to cholestatic injury in different animal models of cholestasis [52,53]. Consistent with an earlier study by Khamphaya et al. [54], ANIT injection resulted in bile duct proliferation.

PCNA is a recognized marker of cell proliferation. It has been widely used to identify proliferating injury of hepatocytes [55]. It was previously reported that Nrf2 signaling activation and NF-κB signaling are accompanied by reduced inflammation-mediated damage and repressed PCNA expression [56,57]. In the current study, ANIT caused a substantial increase in PCNA immunodepression in the liver. In contrast, the number of PCNA-positive cells in the liver was significantly reduced after Uro-B administration, especially with the higher dose. These results suggest that Uro-B suppresses ANIT-induced bile duct proliferation, preventing bile duct damage and, eventually, cholestasis. Such a finding correlated with the previous one and confirmed that Uro-B restrained the expression of PCNA in a mouse model of colorectal carcinoma [58].

## 5. Conclusions

Collectively, this study provides evidence for the hepatoprotective effect of Uro-B against ANIT-induced CLI. Uro-B exerts powerful antioxidant and anti-inflammatory effects mainly via acting simultaneously on Nrf2, NF-κB, and PPARα response pathways, resulting in activated Nrf2 and PPARα antioxidant and anti-inflammatory signaling but suppressed NF-κB pro-inflammatory signaling.

### Future Directions and Limitations

Our next steps would include further preclinical and clinical studies to validate the efficacy of Uro-B and confirm its safety at relatively high doses. A comprehensive assessment of any potential toxicity, especially on the liver and kidneys, at higher doses would strengthen the safety evaluation. Moreover, the optimal dosing and efficacy of Uro-B in patients with CLI will be addressed. Further investigations into the potential link between the beneficial Uro-B effects obtained in this study and reducing ANIT absorption or improving the protective properties of the intestine could provide deeper insights.

## Data Availability

Data will be made available upon reasonable request.
